# Primary Small Bowel Adenocarcinoma Presenting with Duodenal Stricture and Hepatic and Gastroduodenal Artery Encasement: A Case Report

**DOI:** 10.7759/cureus.4534

**Published:** 2019-04-24

**Authors:** Mitchell K Ng, Alexander Lewis, Matthew Lacey, Anaibelith D Perez, Amanda L Pensiero

**Affiliations:** 1 Internal Medicine, Case Western Reserve University School of Medicine, Cleveland, USA; 2 Neurology, University Hospitals Cleveland Medical Center, Cleveland, USA; 3 Internal Medicine, University Hospitals Cleveland Medical Center, Cleveland, USA; 4 Pathology, Louis Stokes Cleveland Veterans Affairs Medical Center, Cleveland, USA

**Keywords:** small bowel cancer, duodenal adenocarcinoma, folfox chemotherapy

## Abstract

Primary small bowel adenocarcinoma is rare, with an estimated U.S. annual incidence of 3.9 cases per million persons, and is often associated with a poor prognosis. We report a case of a 68-year-old male diagnosed with primary duodenal adenocarcinoma with hepatic artery and gastroduodenal artery encasement. The patient initially presented with persistent nausea and vomiting unresponsive to ondansetron and metoclopramide, and initial computed tomography (CT) of abdomen and pelvis revealed significant stomach distension concerning for gastric outlet obstruction. Esophagogastroduodenoscopy (EGD) revealed significant duodenal stricture, with results of triple phase CT of pancreas significant for tissue encasing the common hepatic artery and the origin of the gastroduodenal artery. Pathology results verified the presence of a moderately differentiated adenocarcinoma involving the small bowel. Due to artery encasement by the tumor, the patient was deemed to be a poor surgical candidate, and instead received a duodenal stent for symptomatic relief with initiation of a chemotherapy regimen consisting of folinic acid, oxaliplatin, and fluorouracil (FOLFOX) as an outpatient. This case highlights the presentation and diagnostic workup of a rare cancer.

## Introduction

Small bowel cancers are rare, accounting for less than 5% of gastrointestinal cancers [1]. The four predominant subtypes of small intestinal cancers are adenocarcinomas (30-40%), neuroendocrine tumors (e.g., carcinoid; 35-42%), lymphomas (15-20%), and stromal tumors (e.g., gastrointestinal stromal tumors, angiosarcomas, Kaposi sarcoma) (10-15%) [2]. Annual incidence is estimated at 0.3-2.0 cases per 100,000 people, with higher prevalence in African-Americans and a median age of diagnosis during the 6th decade of life [3]. Among small bowel adenocarcinomas (SBA), the duodenum is the most common site of involvement (55-82%), in contrast to jejunum (11-25%) and ileum (7-17%) [4].

Known environmental risk factors to small bowel adenocarcinomas include alcohol consumption and smoking [5]. It is believed the significant difference in incidence between SBA and colorectal adenocarcinomas is in part due to the decreased exposure time between small intestinal cells and dietary carcinogens relative to the colon, lower microbiota density resulting in decreased xenobiotic transformation, and the presence of microsomal enzymes with potential anti-carcinogenic effects in small bowel epithelial cells [3].

The clinical presentation of SBA is initially non-specific and includes abdominal discomfort, pain, nausea, vomiting, gastrointestinal bleeding and intestinal obstruction. As a result, diagnosis is often delayed by an average of 6-10 months after symptoms appear [3]. One single-center study of 217 patients with SBA revealed diagnosis often occurred in the context of an emergency, most often either an occlusion (40%) or bleeding (24%) [6]. Unfortunately, given the lack of available SBA screening methods and the overall delay in diagnosis, SBA often presents in advanced stages. Current studies have cited that 35-38% of patients present with synchronous metastases and 38-39% of patients present with lymph node invasion [6, 7].

Our case highlights the presentation of a 68-year-old male diagnosed with primary small bowel adenocarcinoma with encasement of the common hepatic and gastroduodenal artery. Given the tumor’s location and artery encasement, surgical resection was not possible. A duodenal stent was placed to alleviate the patient’s stricture, and he was started on folinic acid, fluorouracil, and oxaliplatin (FOLFOX) as an outpatient.

## Case presentation

A 68-year-old African-American male with recent right posterior limb stroke (on daily aspirin 81 mg and clopidogrel 75 mg), chronic kidney disease (CKD) stage III, heart failure with reduced ejection fraction (HFrEF, ejection fraction 35-40%), hypertension (HTN), gout, prediabetes, and hyperlipidemia presented to the emergency department (ED) with persistent nausea and vomiting. He reported initial onset of symptoms six weeks prior to presentation, with nausea and occasional bouts of non-bloody non-bilious emesis. Of note, he had presented to the ED four weeks prior with similar symptoms, and had been discharged with omeprazole 40 mg daily and ondansetron 4 mg as needed for suspected gastroesophageal reflux disease (GERD). Symptoms improved for roughly one week, but gradually returned. He was able to tolerate his meals throughout the day, however reported developing “cramping” abdominal pain around dinner with symptoms exacerbated by laying down, not associated with eating. He reported a three-week history of brown diarrhea (two episodes per day), denying any bleeding (hematochezia, hematemesis, or melena). Per chart review, he had lost ~30 pounds in the past 12 months (213 lbs to 186 lbs on presentation). He denied changes in medications, recent travel, sick contacts, or changes in diet.

Presenting vitals were significant for temperature 97.9 degrees Fahrenheit (normal range: 97.7-99.5 degrees Fahrenheit), pulse 76 (normal range: 60-100), blood pressure 114/71 (normal: 120/80), and respiratory rate 20 (normal range: 12-20). Complete blood count was significant for anemia with a hemoglobin 9.9 g/dL (Hgb 11.9 two months prior, normal range: 13.5-17.5 g/dL for males), and hematocrit 30.9% (normal range: 45-52% for males). Renal function panel (RFP) was remarkable for blood urea nitrogen (BUN) 24 mg/dL (normal range: 7-20 mg/dL), creatinine (Cr) 2.1 mg/dL (normal range: 0.6-1.2 mg/dL), otherwise within normal limits (patient’s baseline renal function was BUN/Cr 14/1.0). Computed tomography (CT) imaging of the abdomen and pelvis without contrast obtained in the ED demonstrated a significantly distended stomach, concerning for potential gastric outlet obstruction with several subcentimeter pulmonary nodules identified bilaterally. Rectal exam revealed heme positive with dark maroon blood. The patient was admitted to medicine for further evaluation.

**Figure 1:**
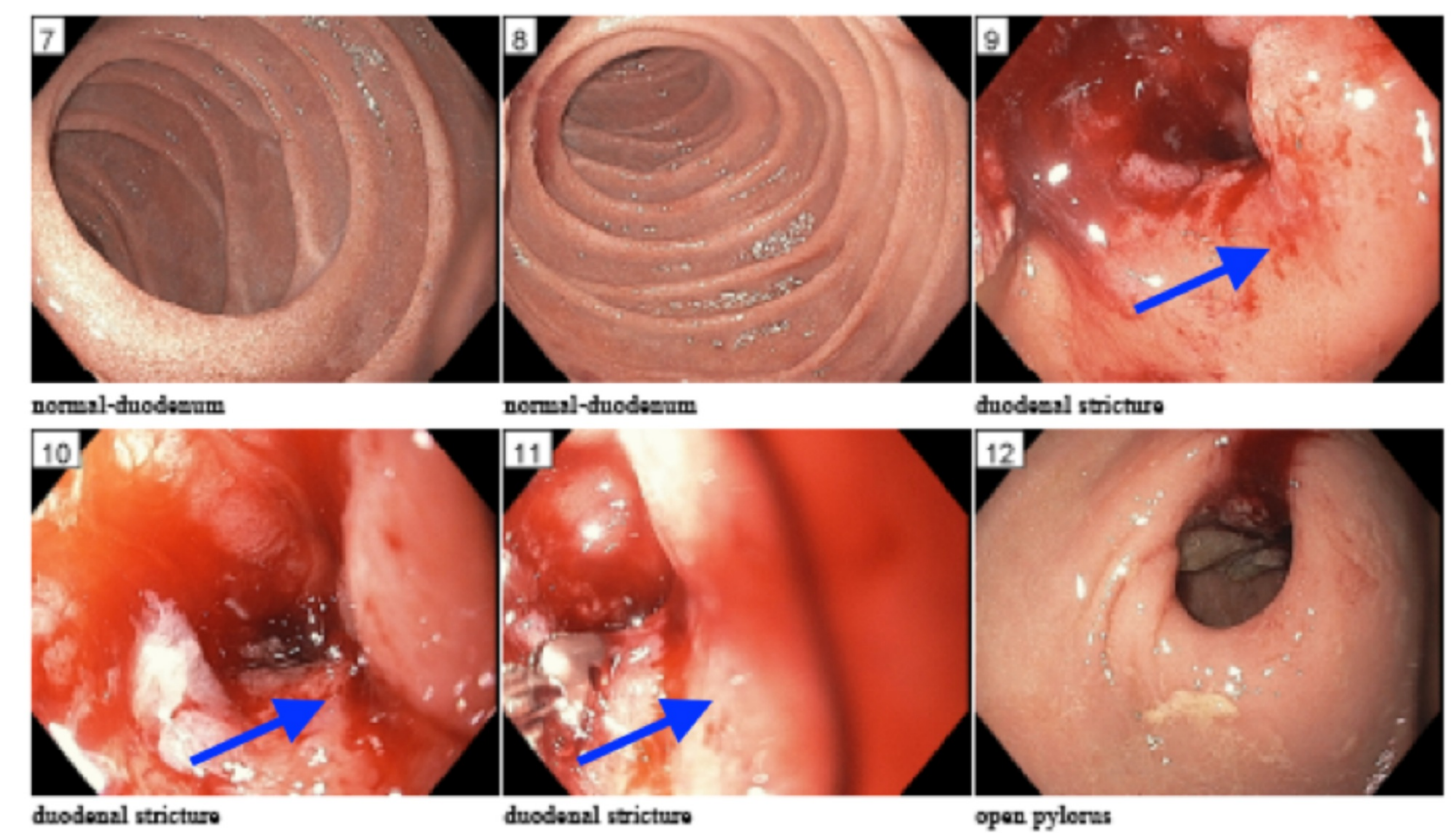
Esophagogastroduodenoscopy (EGD) revealed short inflammatory appearing and traversable stricture at the duodenal sweep (highlighted by blue arrows), area was friable with contact bleeding and multiple cold forceps biopsies were obtained. The second portion of the duodenum appeared to be normal.

**Figure 2:**
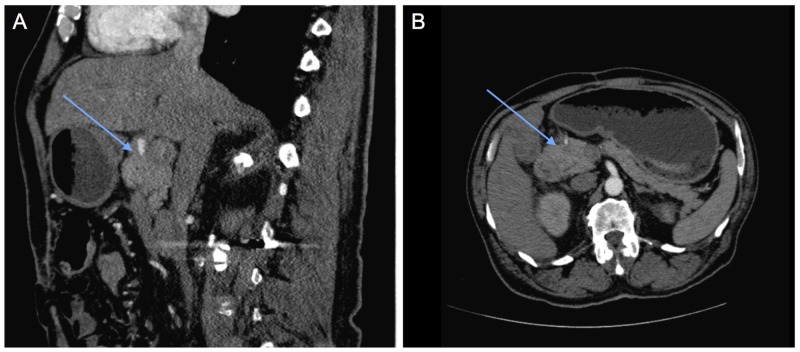
Triple phase computed tomography (CT) pancreas significant for abnormal 7.7 cm x 4.6 cm soft tissue mass encasing common hepatic artery and origin of gastroduodenal artery (as indicated by blue arrows), observable in both (A) sagittal and (B) transverse views.

**Figure 3:**
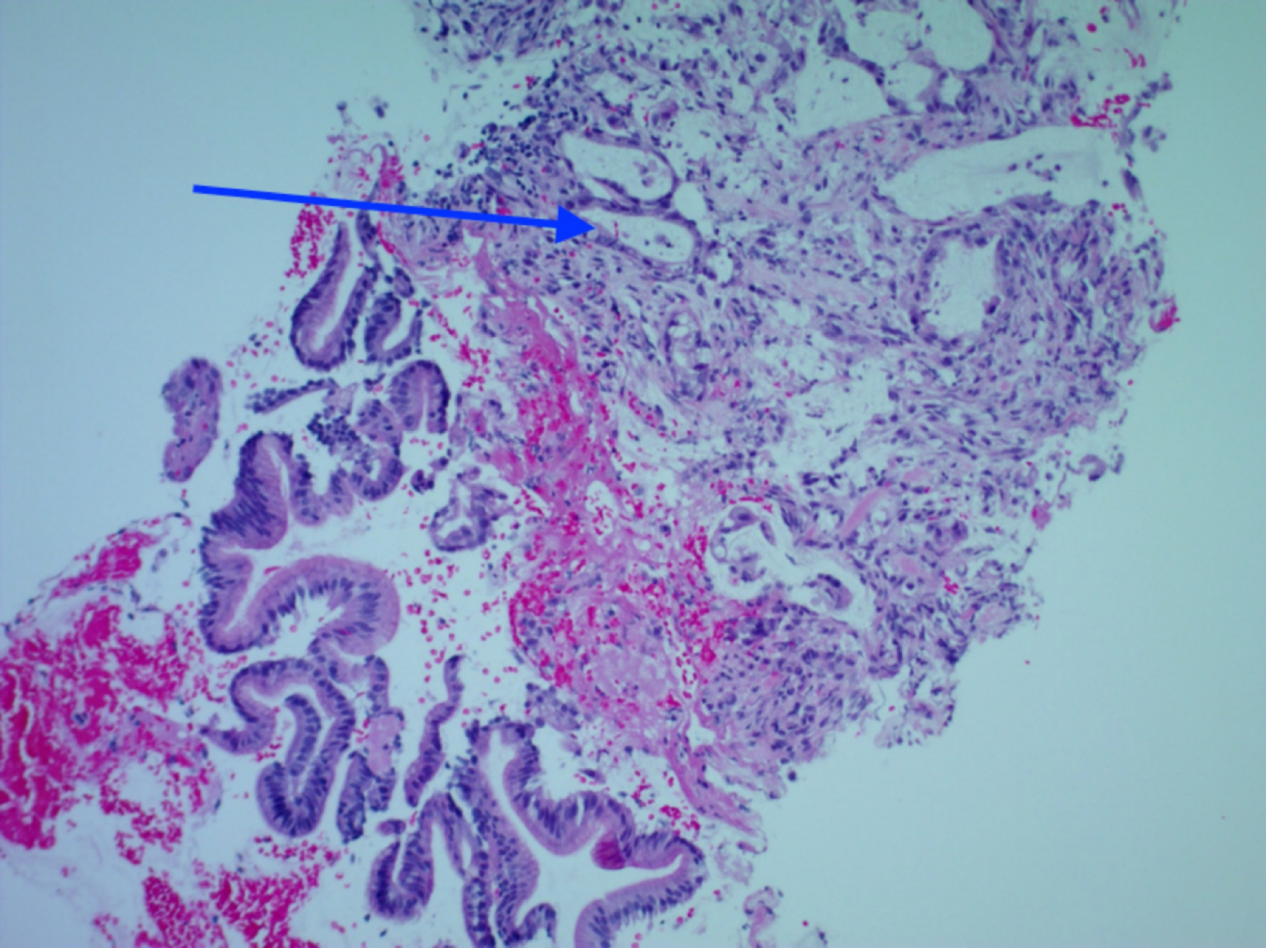
Detached fragments of small intestinal surface epithelium with a fibrotic stroma involved by adenocarcinoma (highlighted by blue arrow).

Due to concern for gastric obstruction, GI performed esophagogastroduodenoscopy (EGD), which revealed a short transversible stricture at the duodenal sweep (Figure 1) and a normal 2nd duodenal portion, with multiple cold forceps biopsies taken. At this time, the differential diagnosis per gastrointestinal (GI) team included peptic ulcer disease (PUD)-associated stenosis (given distension and atrophic stomach mucosa) vs obstruction from adhesion from abdominal surgery (patient had a history of a gunshot wound to the abdomen requiring surgical repair and colostomy in 1979) vs gastroparesis from uncontrolled type 2 diabetes (patient’s hemoglobin A1c was 15.6% in 2013) vs malignancy. Preliminary pathology from EGD biopsy was concerning for malignancy, so triple phase CT of pancreas with and without contrast was obtained for further evaluation. CT results were significant for severe gastric distention with abnormal wall thickening in the proximal duodenum (first and proximal second portion) consistent with a localized mass, specifically abnormal soft tissue 7.7 cm x 4.6 cm encasing the common hepatic artery and the origin of gastroduodenal artery, and punctate/subcentimeter foci of arterial enhancement in the left/right hepatic lobes with a few nonspecific mildly enlarged periportal lymph nodes (Figure 2). Pathology results were significant for moderately differentiated adenocarcinoma involving small bowel, involving mucosa with no intact surface epithelium, and separate fragments of small bowel mucosa uninvolved with no dysplasia (Figure 3). Based on clinical/radiologic correlation, it was later revealed to be a primary small bowel adenocarcinoma.

Surgery for tumor resection was contraindicated given the encasement of the hepatic and gastroduodenal arteries by the duodenal mass. Given the patient's ongoing vomiting in the setting of severe duodenal stricture, gastroenterology performed another EGD with successful placement of a 22 mm, 6 cm long uncovered metal stent placed at the distal duodenal bulb. The day after endoscopy, the patient was tolerating low residual diet well.

The patient's malignancy was unable to be accurately staged, given that suspicious hepatic and lung nodules concerning for metastases were too small to biopsy. Based on their recommendations, the patient was set to receive folinic acid, fluorouracil, and oxaliplatin (FOLFOX) as an outpatient. Prior to discharge, a right internal jugular Mediport was placed under ultrasound/fluoroscopy guidance, a baseline echocardiogram was obtained (EF 35-40% in 12/2017) which revealed intact left ventricular systolic function (EF 50-55%), labs were obtained and revealed an elevated carbohydrate antigen (CA) 19-9 (88 U/mL, normal range: 0-37 U/mL), carcinoembryonic antigen (CEA) within normal limits (1.6 ng/mL, normal range: <3 ng/mL), lactate dehydrogenase (LDH) within normal limits (120 U/L, normal range: 100-190 U/L). Pathology was also sent for Herceptin (Her) 2 and microsatellite instability (MSI) testing. The patient was hemodynamically stable and medically clear for discharge, and instructed to follow up with oncology outpatient for initiation of chemotherapy.

## Discussion

Primary small bowel adenocarcinoma accounts for less than 0.5% of all gastrointestinal malignant neoplasms [3]. The few published studies are retrospective reviews with small sample sizes that cover a period of up to 20 years [4, 6, 8]. As a result, the pathogenesis of SBA is poorly understood. As the majority of SBAs arise from preexisting adenomas which transform into malignant carcinomas, it has been proposed SBAs follow the same sequence as colon cancers [9] and arise from a combination of environmental exposures and a genetic predisposition. For example, SBAs have been associated with increased beta-catenin nuclear accumulation, and K-ras mutations have been found in up to 57% of patients [10].

Given the non-specific clinical presentation such as nausea or weight loss observed in this patient, diagnosis is often delayed. Among imaging studies, CT scans have an overall accuracy of 47% [11] while newer techniques such as CT enteroclysis (sensitivity 85-95%, specificity 90-96%) and capsule endoscopy (sensitivity 89-95%, specificity 75-95%) hold potential for earlier diagnosis [3]. Esophagogastroduodenoscopy remains a key diagnostic test for duodenal adenocarcinomas, detecting up to 88.6% of all tumors [12]. After SBA diagnosis, current guidelines recommend thoraco-abdomino-pelvic CT to assess for potential metastasis, both upper and lower GI endoscopy, and baseline CEA and CA 19-9 (biomarkers for prognostic value), as performed in this patient [13].

Prognosis is poor with reported overall five-year survival rate from 14 to 33% [3, 4, 6, 8]. The most important prognostic factor for SBA is lymph node invasion, while other known poor prognostic factors include advanced age over 75 years, large tumor size, poorly differentiation, lymphovascular invasion, or transmural invasion [6, 8]. Complete surgical resection is currently the only definitive and potentially curative treatment. A French study that included 100 patients diagnosed with SBA who underwent curative surgical resection resulted in five-year overall survival rate of 63% and 52% for SBA without and with lymph node involvement respectively [9]. Of note, in SBA patients presenting with duodenal obstruction who are not surgical candidates, duodenal stents, gastrojejunal bypass and palliative resection may still be considered for symptomatic relief [14].

For patients with unresectable tumors or metastatic disease, chemotherapy is recommended. Several retrospective studies have suggested platinum-based chemotherapy (cisplatin, oxaliplatin, etc.) relative to other chemotherapy regimens carry higher overall response rates (ORR) (46% vs 16%) and longer progression-free survival (PFS) rates (8.7 vs 3.9 months) [15]. Currently, first-line treatment is an oxaliplatin-based chemotherapy regimen such as FOLFOX (combination of folinic acid, oxaliplatin, 5-fluorouracil) or XELOX (oxaliplatin and capecitabine) [15], with FOLFIRI (combination of folinic acid, irinotecan, 5-fluorouracil) as second-line [9, 16]. Nevertheless, SBA patients who are not surgical candidates carry poor outcomes. Earlier diagnostic workup of patients with nonspecific GI symptoms and subsequent surgical resection remain the most important factors in improving outcomes.

## Conclusions

In summary, physicians should be aware of small bowel adenocarcinoma as a rare but important diagnosis to consider in patients who present with persistent non-specific GI symptoms such as nausea and vomiting. Early and prompt diagnosis of SBA with subsequent surgical resection carries the best chance of definitively improving long-term patient outcomes. In our case, surgical resection was not an option given tumor encasement of the common hepatic and gastroduodenal artery. A missed initial diagnosis delayed treatment for four weeks and likely did not change our patient’s outcomes, but underscores the need for clinical providers to carry a higher degree of suspicion and perform a more thorough investigation for earlier diagnosis and treatment of this disease.
